# The complete mitochondrial genome of large odorous frog, *Odorrana graminea* (Amphibia: Ranidae) and phylogenetic analysis

**DOI:** 10.1080/23802359.2019.1674716

**Published:** 2020-08-09

**Authors:** Xiangxiang Jin, Weiye Li, Shijia Hu, Wangming Li, Jianchun Yang

**Affiliations:** a Guangdong Key Laboratory of Animal Conservation and Resource Utilization, Guangzhou, China; b Guangdong Public Laboratory of Wild Animal Conservation and Utilization, Guangzhou, China; c Guangdong Institute of Applied Biological Resources, Guangzhou, China

**Keywords:** Complete mitochondrial genome, *Odorrana graminea*, sequence analysis

## Abstract

The mitochondrial genome of *Odorrana graminea* was sequenced and analyzed. The complete mitochondrial genome is 18,106 bp in length, including 13 protein-coding genes (PCGs), 2 ribosomal RNA genes, 21 transfer RNA genes, and a control region (D-loop). All PCGs are initiated by ATN codons, except for GTG for *COI* and *ND5*. Five PCGs use a common stop codon of TAA or TAG, whereas *COI* terminats with AGG as stop codon; *ND6* with AGA; the other six ends with an incomplete stop codon (a single stop nucleotide T). The analysis results based on Bayesian inference method provide a useful resource for the phylogenetic studies of superfamily Ranoidea.

The large odorous frog *O. graminea* is an arboreal, nocturnal frog living near noisy streams and waterfalls in mountainous areas of southern China (Shen 2018). However, *O. graminea* is threatened by human activities, such as habitat destructions, excessive captures, and pollutions (Fei et al. 2012). Therefore, it has been listed in The IUCN Red List (https://www.iucnredlist.org/species/58608/11808913). In this study, we determined the complete mitochondrial DNA (mtDNA) sequence of *O. graminea* (GenBank accession no. MN417331) for the protection of this species.

The specimen of *O. graminea* was collected from Guanshan National Nature Reserve in Yifeng County, Jiangxi Province, China (N28°30′–28°40′, E114°29′∼114°45′), and was deposited in Guangdong Institute of Applied Biological Resources (No. 2018098). Total genomic DNA was extracted with the TaKaRa MiniBEST Universal Genomic DNA Extraction Kit (Takara, Beijing, China) following the kit’s instructions. The high-quality genomic DNA were generated on an Illumina HiSeq4000 using sequencing protocols provided by the manufacturer (Illumina, Inc., San Diego, CA, USA).

The complete mitochondrial genome of *O. graminea* is 18,106 bp in length with 29.40% A, 27.96% C, 14.75% G, and 27.89% T. The genome is comprised of 13 protein-coding genes, 2 ribosomal RNA genes, 22 transfer RNA genes, and 1 control region, similar to typical vertebrate mtDNA. The total length of 22 tRNA genes is 1523 bp, ranging from 64 bp (*tRNACys*) to 73 bp (*tRNALeu* (UUR) and *tRNAAsn*). The total length of 13 protein-coding genes is 11,295 bp. The start codons are ATG for *ND1*, *COII*, *ATP8*, *COIII*, *ND3*, *ND4L*, *ND4*, *ND6*, and *CYTB* while ATT for *ND2*, GTG for *COI* and *ND5* and ATA for *ATP6*. *COI* terminats with AGG as stop codon; *ND6* with AGA; *ATP*8, *ND4*L, *ND5* and *CYTB* with TAA; *ND4* with TAG, and other six PCGs end with an incomplete stop codon (a single stop nucleotide T). The two ribosomal RNA genes including *12SrRNA* (933 bp) and *16SrRNA* (1585 bp) are commonly located between *tRNAPhe* and *tRNALeu* (UUR). The control region is 2568 bp in length with 63.86% A + T content. Within the complete mitochondrial genome of *O. graminea*, there are five reading frame overlaps (The *tRNAGln* and *tRNAMet* share one nucleotide; OL and *tRNACys* share three nucleotides; *COI* and *tRNASer* share nine nucleotides; *ATP8* and *ATP6* share one nucleotide; *ND4L* and *ND4* share seven nucleotides).

The phylogenetic analysis of the superfamily Ranoidea was conducted with other 14 Ranoidea mitogenomes and *Leptolalax oshanensis* (Xiang et al. 2013) mt genome as the outgroup with Bayesian inference (BI). The best-fitted substitution model was determined in MUSCLE v.3.8.31 (Edgar 2004) by Akaike Information Criterion (AIC). The BI was conducted in MrBayes 3.2.6 (Ronquist et al. 2012). As shown in Figure 1, the phylogenetic positions of these 16 mt genomes are successfully resolved with high bootstrap supports. The result indicates *O. graminea* and *O. schmackeri* (Bu et al. 2016) are closest and the genetic distance between Ranidae and Mantellidae are closer than others.

**Figure 1.**
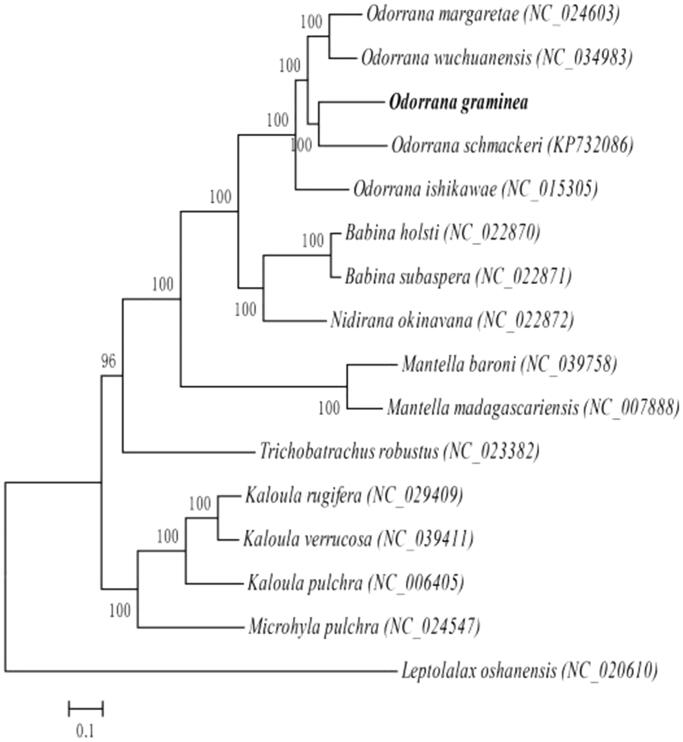
Phylogenetic relationships (Bayesian inference) among the species of the superfamily Ranoidea based on the mitochondrial genome nucleotide sequence of the 13 protein-coding genes. Numbers beside the nodes are percentages of 1000 bootstrap values. *Leptolalax oshanensis* was used as outgroup.
